# Neural and behavioral investigations into timbre perception

**DOI:** 10.3389/fnsys.2013.00088

**Published:** 2013-11-13

**Authors:** Stephen M. Town, Jennifer K. Bizley

**Affiliations:** Ear Institute, University College LondonLondon, UK

**Keywords:** auditory cortex, vowels, ferret, speech, neural coding

## Abstract

Timbre is the attribute that distinguishes sounds of equal pitch, loudness and duration. It contributes to our perception and discrimination of different vowels and consonants in speech, instruments in music and environmental sounds. Here we begin by reviewing human timbre perception and the spectral and temporal acoustic features that give rise to timbre in speech, musical and environmental sounds. We also consider the perception of timbre by animals, both in the case of human vowels and non-human vocalizations. We then explore the neural representation of timbre, first within the peripheral auditory system and later at the level of the auditory cortex. We examine the neural networks that are implicated in timbre perception and the computations that may be performed in auditory cortex to enable listeners to extract information about timbre. We consider whether single neurons in auditory cortex are capable of representing spectral timbre independently of changes in other perceptual attributes and the mechanisms that may shape neural sensitivity to timbre. Finally, we conclude by outlining some of the questions that remain about the role of neural mechanisms in behavior and consider some potentially fruitful avenues for future research.

## Introduction

Timbre is operationally defined as the attribute that distinguishes sounds of equal pitch, loudness, location and duration. Functionally, timbre is a key determinant of sound identity, and plays a pivotal role in speech as it is the principal determinant of phonetic identity. Despite its importance, timbre remains one of the least studied and perhaps most challenging features of sound to understand. To systematically study timbre, it is necessary to relate the acoustic differences between sounds to their perceived timbre both in human listeners and in species that may form suitable animal models for studying the neural basis of timbre perception at the single cell level. Here, we summarize the primary acoustic features thought to underlie timbre perception in humans and discuss evidence demonstrating that animals can perceive and discriminate these features in a similar fashion. We then explore the suggestion that timbre is an important component of the vocalizations of many species and thus has a general ecological significance in animal communication. We then review our current understanding of the representation of timbre in the brains of both human and non-human listeners.

## The psychoacoustics of timbre perception

### Timbre in speech

Speech perception involves the perception of many sound attributes including dynamic patterns of pitch, loudness and timbre changes. Speech signals contain a wide variety of acoustic cues from which sound timbre may be derived and our perception of any one segment of speech may be influenced by the context in which it occurs. At the phonetic level, timbre plays a crucial role in determining the identity of vowels and consonants.

**Figure 1:**
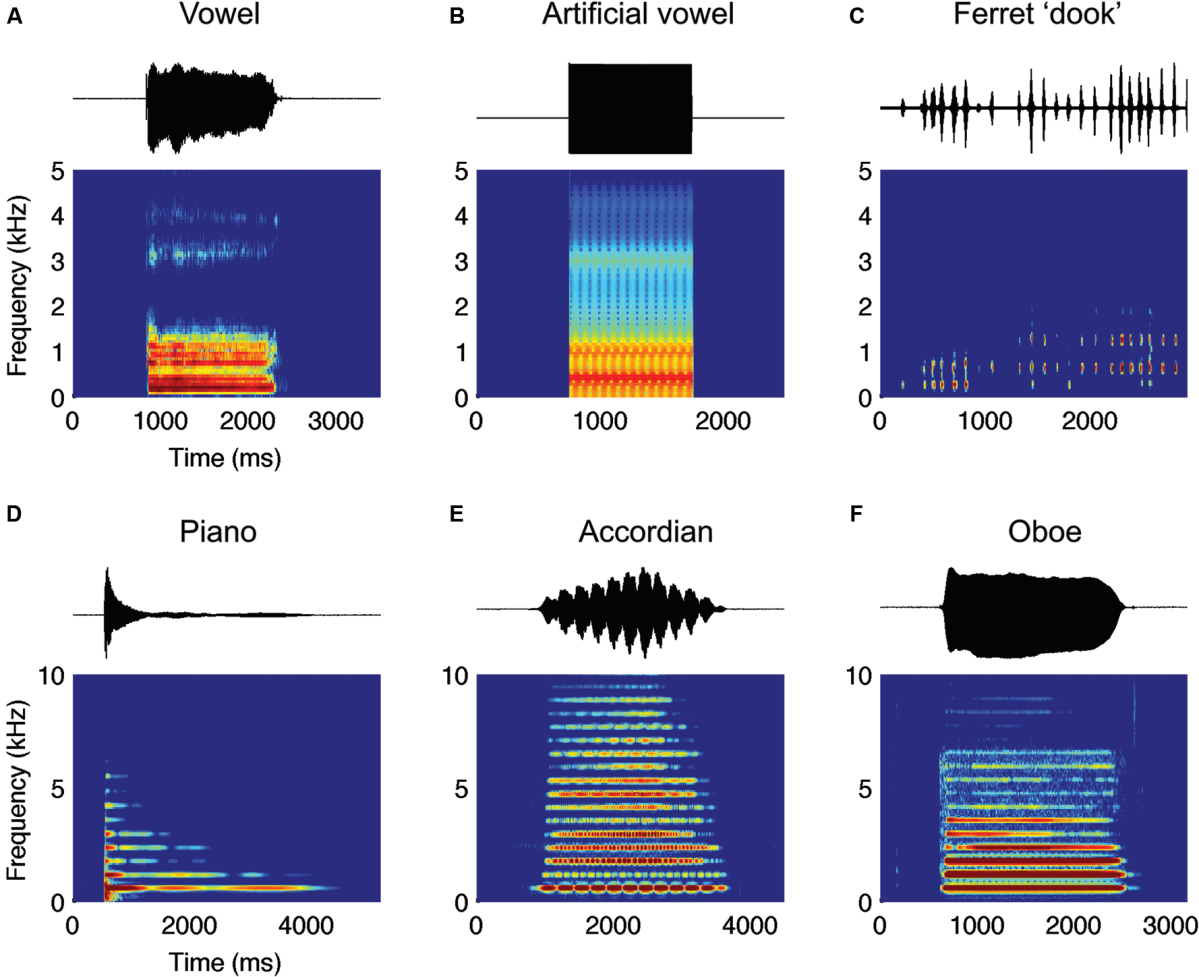
**(A)** Amplitude waveforms (top) and spectrograms (bottom) for a female voice speaking “a” (as in “hard”) **(B)** an artificial “a” and **(C)** a male ferret making a series of “dook” calls. Such calls have a harmonic structure. (D–F) Amplitude waveform and spectrograms for **(D)** Piano, **(E)** Accordion and **(F)** Oboe, playing the same note. Note that although all three have the same fundamental frequency (and therefore pitch) the relative distribution of energy across the harmonics differs, enabling each to have a characteristic timbre. Also important is the shape of the temporal envelope—each has a different onset dynamic and the characteristic vibrato of the accordion is clearly evident in the amplitude waveform.

Analysis of spoken phonemes, and playback experiments with synthesized speech sounds, indicate that formants play a critical role in the perception of vowel and consonant timbre. Formants are peaks in the steady-state frequency-amplitude spectrum (Figure 1A, for a natural vowel and Figure 1B for an artificially generated vowel) introduced by the resonant properties of the vocal tract. Formant distributions can be characterized by a variety of summary statistics including the position of formant peaks, formant amplitude and bandwidth. Historically, formant positions have been proposed to play the principal role in determining vowel identity. Spoken vowels form clusters according to phonetic identity within a space defined by the location of the first (F1) and second formants (F2; Potter and Steinberg, 1950; Peterson and Barney, 1952). The distribution of F1 and F2 positions of spoken vowels matches the distributions of first and second components identified by principal components analysis of vowel spectra (Plomp et al., 1967). When vowels were synthesized, variation in formant positions can be sufficient for discrimination and identification of vowels (Delattre et al., 1952; Klatt, 1982; Molis, 2005; Swanepoel et al., 2012) and perturbation of formant positions distorts both psychophysical and phonetic judgments of vowel similarity (Carlson et al., 1979; Klatt, 1982). Furthermore, introducing spectral notches or masking noise close to formant peaks affects listener’s perception of, and discrimination between, vowels (Pickett, 1957; Carlson et al., 1979; Kasturi et al., 2002; Swanepoel et al., 2012). However, a model of vowel timbre perception based solely on formant position would be incomplete as it is sometimes possible to discriminate vowels with similar formant positions (Bladon, 1983; Sakayori et al., 2002). It is likely that such discrimination involves additional features of the spectrum such as the amplitude and bandwidth of formants, if not the entire spectral shape introduced by vocal tract filtering (Christovich and Lublinskaya, 1979; Dubno and Dorman, 1987; Beddor and Hawkins, 1990; Ter Keurs et al., 1992; Zahorian and Jagharghi, 1993; Ito et al., 2001; Molis, 2005). The dispersion of formants, that is the average distance between adjacent formant peaks, also indicates the size of a talker as formant dispersion is closely correlated with vocal tract length (Fitch, 1997), which is in turn correlated with body size of humans (Fitch and Giedd, 1999; Smith et al., 2005).

Formants also play a significant role in consonant perception. Consonant identity depends on movement in formant peak position over time known as formant transitions (Liberman et al., 1967; Lisker, 1986) and in particular transitions of the second and third formants (Liberman et al., 1954; Harris et al., 1958; Li et al., 2010). Formant transitions follow an initial noise burst resulting from the release of constriction within the vocal tract. (This constriction distinguishes consonants from vowels in which the vocal tract is fully open). The frequency content of the initial burst and its temporal envelope can also act as cues to consonant identity (Liberman et al., 1967; Li et al., 2010). Formant position may also vary over the duration of a vowel (Hillenbrand et al., 1995), and although these formant movements are slower and smaller than formant transitions, listeners more accurately identify synthesized vowels when the natural formant movements are present than when they are removed (Hillenbrand and Nearey, 1999; Assmann and Katz, 2000, 2005).

To summarize briefly, many spectral and temporal features of sound may give rise to timbre in vowels and consonants, with the potential for a large degree of redundancy. The relative importance of acoustic cues determining vowel/consonant identity may not be fixed, but rather may vary depending on the linguistic experience and environmental conditions of the listener as well as the phonetic context and the individual speaking. Understanding the acoustic basis of timbre in speech is thus a complex problem. When designing balanced experiments for humans and other animals, this difficulty is emphasized by the high dimensionality of spectral and temporal cues. As we will discuss in Section Animal Models of Timbre Perception, in these cases summary statistics such as formant positions can provide a useful low-dimensional parameter space in which to systematically investigate neural processes underlying timbre perception.

### Musical and environmental timbre

Timbre also distinguishes musical notes of the same pitch, loudness and duration played by different musical instruments (e.g., an oboe and violin). The underlying features of sound that contribute to differences in perceived timbre can be investigated by comparing the acoustic properties of instruments. Such comparisons indicate that, like vowels and consonants, both spectral and temporal features of sound determine timbre. In the spectral domain, most musical instruments emit harmonic resonances, that is, they produce energy at integer multiples of a fundamental frequency (the harmonics are evident as the horizontal bands in the spectrograms in Figure 1). Such harmonics resemble those introduced in speech by the vibration of the vocal chords. As with vowels, the distribution of energy across different harmonics is one of the key differences between different musical instruments. For example, the piano (Figure 1D) has sustained energy only at the fundamental while the violin and accordion (Figure 1E) has energy distributed over many harmonics, and the oboe contains most of its energy in the first five harmonics (Figure 1F). Some instruments, such as the clarinet, have energy only in the odd harmonics, whereas notes played by the trombone only have energy at the first and second harmonic (Campbell and Greated, 1994). The second key determinant of the timbre of a musical instrument is its temporal characteristics, or what musicians call the “nature of attack”. This is especially the case for plucked string instruments like the harp, or piano whose notes contain little or no steady state sound at all (Figure 1C; Campbell and Greated, 1994). In this case, the shape of the amplitude envelope at the beginning of the sound will be key to the perceived tone quality.

The acoustic basis of musical timbre has also been studied using multidimensional scaling (MDS) techniques (Plomp and Steeneken, 1969; Miller and Carterette, 1975; Grey, 1977; Wessel, 1979; McAdams, 1999; McAdams and Giordano, 2009). Simply put, MDS aims to determine the components and underlying structure of a data space from a series of distance measures. Here, the space of interest is the perceptual representation of musical timbre and the distance measures are dissimilarity judgments of listeners to instrument sounds. After constructing a timbre space using MDS, it is possible to relate different perceptual dimensions back to the acoustic features of the instrument sounds. For example, Grey (1977) found that similarity judgments of synthesized instrument sounds could be mapped into a three-dimensional space in which dimensions were strongly correlated with spectral shape, the presence of low-amplitude high-frequency energy in the attack segment of sounds and the combination of spectral fluctuation with synchronous high frequency transients. Numerous MDS studies since have replicated the finding that the spectral shape of instrument sounds (or related statistics such as spectral centroid) and attack time are important components of timbre spaces (McAdams et al., 1995; Lakatos, 2000; Caclin et al., 2005; Burgoyne and McAdams, 2008). The hypothesized roles of spectral shape and attack time are also consistent with changes in perception following stimulus manipulation. Specifically, switching of the spectral shape of synthetic tones leads to systematic changes in the position of stimuli within a perceptual space generated by MDS (Grey and Gordon, 1978). Similarly, sound onsets containing the attack phase are sufficient (Iverson and Krumhansl, 1993) and in some cases necessary for optimal identification of musical instruments (Berger, 1964; Wedin and Goude, 1972). In addition to the contribution of spectral and temporal features, recent work (Elliott et al., 2013) has suggested that joint distributions of spectrotemporal features influence instrument timbre. Timbre not only enables musical instrument identification, but also enables listeners to estimate the scale of an instrument. In addition to listeners being able to recognize the family of an instrument sound, even when that sound was modified in pitch and scale beyond the range normally experienced, listeners could accurately assess the scale of a given instrument (van Dinther and Patterson, 2006).

Finally, timbre also plays a role in the perception of environmental sounds such as impacted bars or plates, that is, sounds produced when a bar or plate is struck. Human listeners are able to classify the material, hollowness and, to a lesser extent, size and shape of such bars or plates from the impacted sound alone (Lakatos et al., 1997; Kunkler-Peck and Turvey, 2000; Lufti, 2001; Tucker and Brown, 2003; Lufti, 2007). The classification of a bar or plate’s material may depend on its damping properties—the extent to which oscillations in the plate or bar are reduced in amplitude over time after being struck. For example metal and glass plates differ in the extent to which they are subject to thermo- and viscoelastic damping (Chaigne and Lambourg, 2001). When the sounds of impacted plates are artificially damped by suspension in water, listener’s judgments of material, shape and size become less reliable (Tucker and Brown, 2003; although see also Giordano and McAdams, 2006). Damping properties of synthesized impacted bars and plates are associated with sound duration, but also with acoustic features such spectral centroid that are associated with the timbre of vowels and musical instruments (McAdams et al., 2004, 2010). It therefore seems likely that perception of timbre contributes, at least in part, to the classification of materials based on damping properties.

Thus timbre is a significant component of sounds outside of speech. The acoustic cues underlying timbre perception are complex, with both spectral and temporal features generating variations in the perceived timbre of resonant sources. Descriptors such as spectral centroid and attack time in music, of formant position in speech can successfully summarize high-dimensional cues such as spectral shape or temporal modulation whilst also accounting, at least in part, for the influences of those high dimensional cues on timbre perception.

### Animal models of timbre perception

Animal models can contribute to our understanding of timbre perception in several important ways. Firstly, timbre perception is unlikely to be unique to humans and so determining the ability of animals to perceive timbre should elucidate the evolutionary history of our auditory abilities. Secondly, timbre is often considered in relation to sounds generated by humans (i.e., speech and music); however animal studies may reveal a broader role for timbre as a general feature of vocal communication. Correspondingly, the sensitivity of species to sound attributes beyond our own perception may extend models of timbre to more fundamental psychophysical principles. Most importantly, animal models provide the opportunity to apply techniques and experimental designs that would be unfeasible for human studies.

Given the importance of timbre in speech perception, it is perhaps unsurprising that most studies of timbre discrimination in animal models have used spoken or synthetic vowels. Many species, both closely related and evolutionarily distant to humans, are capable of discriminating between vowels. These include other primates such as chimpanzees (Kojima and Kiritani, 1989), baboons (Hienz and Brady, 1988; Hienz et al., 2004), Japanese and rhesus macaques (Dewson et al., 1969; Sinnnott, 1989; Sinnott and Kreiter, 1991; Sommers et al., 1992; Sinnott et al., 1997), Sykes’ and vervet monkeys (Sinnnott, 1989; Sinnott et al., 1997). Vowels can also be discriminated by a range of other mammals including carnivores such as cats (Dewson, 1964; Hienz et al., 1996, 1998; May, 2003), dogs (Baru, 1975) and ferrets (Walker et al., 2011; Bizley et al., 2013); and rodents such as gerbils (Sinnott and Mosqueda, 2003; Schebesch et al., 2010), rats (Eriksson and Villa, 2006) and chinchillas (Burdick and Miller, 1975; Kuhl and Miller, 1975, 1978). Several bird species such as mynahs (Klatt and Stefanski, 1974), parrots and budgerigars can mimic human speech, indicating that they are capable of identifying and reproducing vowel timbre. Additional studies have demonstrated that in psychophysical tasks, blackbirds, cowbirds, pigeons (Hienz et al., 1981) and zebra finches (Ohms et al., 2010, 2012) are capable of discriminating between vowels. Thus acoustic features that distinguish vowels in human speech are audible to species other than humans. We discuss below whether humans and non-humans use the same acoustic features in vowel discrimination and if so, whether these acoustic features are used in the same way.

Formant frequencies are critical cues in the identification and discrimination of vowels by humans and, as summary statistics, provide an experimentally tractable model for studying the spectral basis of timbre perception in animals. MDS analysis has been used to identify the position of the first and second formants as critical determinants of vowel dissimilarity in humans (Pols et al., 1969). MDS approaches have also indicated that formants are important in animal’s perception of vowels (Kojima and Kiritani, 1989; Dooling and Brown, 1990; Sinnott et al., 1997). In such studies, which employ a go/no-go design, subjects are required to detect a change in an on-going sequence of vowel sounds. Both the ability of the subject to detect a change and the speed with which they do so are used as indirect measures of the animal’s perception. Response time may be taken as an indicator of perceptual similarity, i.e., the longer it takes a subject to identify a change from one vowel to another, the more similar the perception of those vowels is thought to be. For chimpanzees (Kojima and Kiritani, 1989), response latencies to detect changes in identity of vowels with similar F1 but distinct F2 positions were longer than for vowels with similar F2 but distinct F1 positions. This suggests that, for these animals, vowels with similar F1 positions were perceptually closer than vowels with similar F2 positions, and thus that vowel perception by chimpanzees places greater weight on the position of the first than second formant. The opposite was true for human subjects performing the same task: response latency was longer to detect changes in vowels with similar F2 but distinct F1 positions than for vowels with similar F1 but distinct F2 positions. Thus humans placed greater weight on the position of the second than the first formant when detecting changes in vowel identity so that vowels with little difference in F2 were hard to discriminate. The distinction between humans and non-human primates in the weighting of first and second formants has also been found when comparing humans, macaques and Sykes’ monkeys (Sinnott et al., 1997). In each species, dimensions of perceptual space could be correlated with formant frequencies. However the weighting of dimensions differed between species: humans weighted F2 position more than F1 position whereas Sykes’ monkeys weighted each formant equally and macaques gave greater weight to F1 than F2.

**Figure 2:**
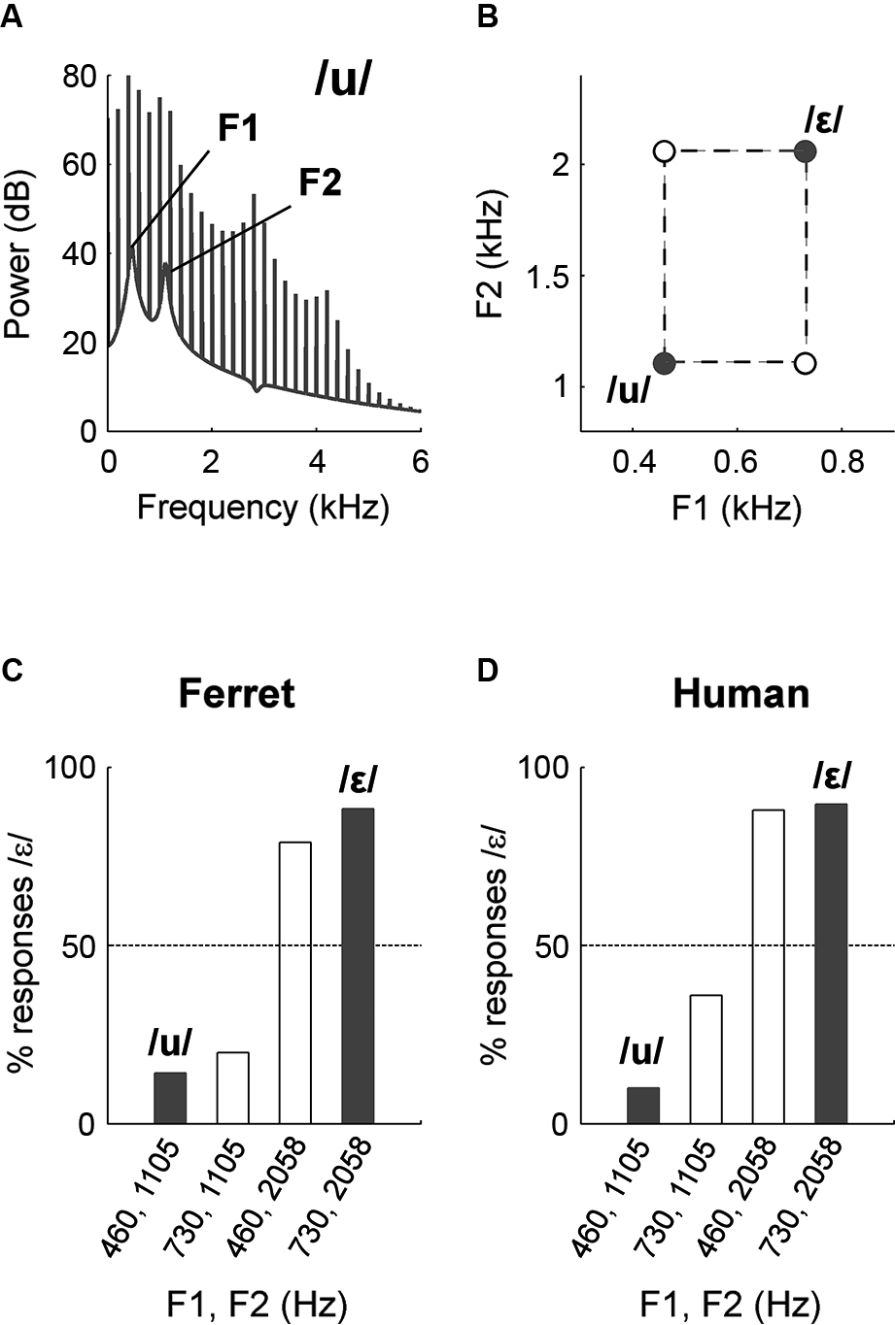
**(A)** The position of the F1 and F2 peaks in the spectral envelope of a synthetic vowel /u/. **(B)** Formant space indicating the position of vowels used to measure the relative contributions of F1 and F2 in vowel identification. Filled circles indicate vowels with which subjects were trained in a 2AFC task. Open circles indicate mismatch vowels presented as probe trials. **(C)** Responses of one ferret to training (filled bars) and probe vowels (unfilled bars). **(D)** Responses of one human to training (filled bars) and probe vowels (unfilled bars).

Humans are not unique in weighting the second formant strongly in vowel identification. Ohms et al. (2012), trained zebra finches in a go/no-go task to respond to one synthetic vowel (S+) and withhold responding to another vowel that differed in F1, F2 and F3 values (S−). Probe vowels were then presented in which the F1 and combination of F2 and F3 were taken from a mismatch of S+ and S−. The correct response to such a probe is ambiguous for the subject (although probes were not rewarded or punished), but the choice made indicates the relative weighting of F1 and the F2–F3 combination. In this case the response of zebra finches was found to be more dependent on the F2–F3 combination than the F1 position of probe stimuli. Thus if a probe stimulus shared the F2–F3 positions of S+, the animal was more likely to respond whereas if the probe stimulus shared the F2–F3 positions of S−, the animal was more likely to inhibit responding. Humans acted similarly in an analogous task in the same study. We have found a similar dependence on F2 in vowel identification by ferrets (Town et al., in preparation): We trained ferrets in a two-alternative forced choice (2AFC) task to identify synthesized vowels that differed in F1 and F2 (Figures 2A, B). Subjects were then presented with probe sounds in which F1 and F2 positions of training vowels were mismatched (Figure 2B). We found that ferrets responded to probe sounds in the same way as they responded to training vowels with the same F2 value (Figure 2C). For example, if the ferret was presented with a probe vowel that shared the same F2 value as the vowel /ε/ (2058 Hz), then it would respond as if presented with /ε/. Human listeners tested in the same way showed a similar pattern of behavior (Figure 2D).

Why is it that ferrets and zebra finches resemble humans in their weighting of formants whilst non-human primates such as chimpanzees and macaques do not? One answer may lie in the relative sensitivity of each species to sounds within the frequency range of F1 (0.3–1 kHz) and F2 (1–3 kHz). In a typical audiogram, the sensitivity of a human listener increases from F1 to the F2 frequency regions (ISO, 2003). A similar pattern of frequency tuning is seen in ferrets (Kelly et al., 1986) and zebra finches (Okanoya and Dooling, 1987). In contrast, audiograms for chimpanzees and macaques have equal or higher thresholds around the F2 than F1 region of human vowels (Behar et al., 1965; Kojima, 1990; Coleman, 2009) making these animals more relatively sensitive to sound within the F1 frequency region. It should be noted that methodological differences between audiogram measurements exist between species that confound this suggestion at present. Audiograms for primates were measured using headphones (closed-field) whereas measurements for non-primates were made using speakers (open-field). Meta-analysis of primate data (Coleman, 2009) suggests that it is difficult to compare audiograms measured using open and closed field methods. In future, it will be necessary to resolve these methodological differences to confirm the extent to which formant weighting can be accounted for by frequency sensitivity illustrated in audiograms.

Sensitivity to changes in formant positions has also been systematically studied in both humans and animals. For changes in frequency (∆F) of the F1 and F2, Kewley-Port and Watson (1994) found thresholds for well-trained humans to be between 1.5% and 2% when expressed as the Weber fraction (∆F/F). Measurements vary between animals but are typically within a comparable range: Human thresholds resemble those measured in macaques (1.6%) when discriminating single formant vowels (Sommers et al., 1992) and in baboons (3%) when detecting changes in the position of the second formant within multi-formant vowels (Hienz et al., 2004). In a direct comparison between species, Sinnott and Kreiter (1991) found that difference limens for detecting changes in F1 and F2 frequency were two to three times greater in macaques than humans. For comparison, thresholds for frequency discrimination of pure tones are at least four times higher in macaques than humans and can be up to twenty times higher at high signal levels (Sinnott et al., 1987). Cats are also more sensitive to changes in formant frequency than pure tone frequency. Thresholds for changes in formant frequencies of synthetic vowels are 2.3% (Hienz et al., 1996) whereas thresholds for pure tone frequency changes are between 6% and 7% (Hienz et al., 1993). This contrasts with the case for humans, where sensitivity to pure tone frequency changes is greater than for formant frequency changes (Wier et al., 1977; Kewley-Port and Watson, 1994). That humans and animals have similar sensitivity to formant frequency changes but not pure tone frequency changes may in part result from superior frequency resolution of the human cochlea, but is also likely to reflect the more ethologically relevant nature of spectrally rich sounds over pure tones and suggests that vowels are well suited for comparative psychoacoustics.

Not only are many animals able to discriminate vowels, but several species have been shown to do so in noisy conditions. Macaques can discriminate between /i/ and /u/ in noise with a signal-to-noise ratio (SNR) of between −11 and −18 dB for vowels presented at 70 dB Sound Pressure Level (SPL; Dewson, 1968). Cats can discriminate vowels in noise presented at 50 dB SPL with an average SNR of −12.3 dB (Hienz et al., 1996). Ferrets can discriminate between /u/ and /ε/ in white noise and low-pass noise presented at 65 dB SPL with a SNR of −10 to −15 dB (Bizley et al., 2013). Threshold discrimination by animals at such noise levels mirrors performance in humans where vowel discrimination is impaired but still possible at an SNR of −10 dB for vowels presented at 70 dB SPL (Swanepoel et al., 2012). Furthermore in both humans and cats, discrimination of vowels in noise is influenced by the SNR at the positions of the first and second formants of vowels (Hienz et al., 1996; Swanepoel et al., 2012). These parallels in vowel discrimination further emphasize the utility of animal models and support the suggestion that mechanisms of timbre discrimination by humans and non-humans overlap considerably.

Finally, a key feature of human vowel perception is perceptual constancy, or invariance, also known as speaker or vocal tract normalization. This is the ability to identify vowels as the same when produced by different speakers, despite variations in acoustic features such as fundamental frequency. In a change detection task, chimpanzees treated vowels spoken by male and female speakers as the same, indicating that they are able to generalize across speakers (Kojima and Kiritani, 1989). Similarly zebra finches can generalize vowel identity across individual speakers of one or both genders (Ohms et al., 2010) while gerbils have been shown to generalize vowel identity across vocal tract length (Schebesch et al., 2010). Thus non-human species can normalize for acoustic features such as pitch that may vary between speakers of the same vowel.

In conclusion, a variety of animals, both phylogenetically close and distant, can discriminate human vowels when spoken or synthesized. Many species are sensitive to the spectral properties of vowels that are important for human perception such as formant frequencies. Species such as the ferret and zebra finch resemble humans in their weighting of individual formants while non-human primates do not. Such species differences/similarities may relate to the pattern of frequency sensitivity observable in animal audiograms. Species such as the ferret and zebra finch thus provide excellent models for the neural basis of timbre perception. Practically speaking, their size and readiness to perform behavioral tasks makes it possible to simultaneously examine perception and the activity of single neurons in well controlled acoustic environments. In the future it will be necessary to extend the study of timbre perception in animal models beyond formant positions to include those spectral and temporal features of sound such as formant movement or spectral shape discussed earlier that also influence vowel timbre. Ultimately it will be necessary to investigate whether acoustic features underlying timbre in human vowels also contribute to the natural vocalizations of the animals under investigation. As we will discuss below, there is evidence that formants and formant-like spectral prominences exist within non-human vocalizations and play a significant role in the behavior of many species. However for candidate models such as the ferret, data on the acoustic features of vocalizations require further investigation.

### Timbre in the natural world

In this section we consider to what extent spectral timbre discrimination is applicable beyond human speech. It is perhaps unsurprising that animals can perceive and discriminate timbre in human speech as the vocalizations of many animals contain spectral prominences that resemble formants in human vowels and thus may provide a timbre to conspecific calls. Examples include, but are not limited to, birdsong (Nowicki, 1987; Cynx et al., 1990), koala bellows (Charlton et al., 2011), audible cries of rodents (Roberts, 1975), deer roars (Reby and Mccomb, 2003), panda bleats (Charlton et al., 2009), ferret dooks (Figure 1C) and a range of primate vocalizations (Andrew, 1976; Fitch, 1997; Harris et al., 2006; Ghazanfar et al., 2007).

In human speech, formants are (by definition) the result of resonant filtering of the vocal tract. Several lines of evidence suggest that the spectral prominences seen in certain animal vocalizations may also result from vocal tract filtering. Experiments using helium-oxygen (heliox) environments have shown that spectral prominences found in birdsong shift significantly when the speed of sound is increased, while the fundamental frequency of calls remains relatively constant (Nowicki, 1987). This separation, a result of the independence of source (syrinx) and filter (vocal tract), supports the suggestion that spectral prominences are introduced by the supra-syringeal apparatus and thus also fulfill the definition of formants. Where heliox experiments are impractical, the involvement of vocal tract filtering has been inferred through a number of approaches (see Fitch and Fritz, 2006 for review). These include the correlation of formant frequencies with vocal tract length or other measures of body size such as height (Fitch, 1997; Riede and Fitch, 1999; Fitch and Kelley, 2000; Reby and Mccomb, 2003) or observed movements of the vocal tract during vocalization (Harris et al., 2006). The role of vocal tract filtering in animal calls suggests that formants are not limited to human speech and therefore that acoustic features associated with the timbre of human vowels may also influence an animal’s perception of conspecific vocalizations.

It has been shown that animals are sensitive to shifts in formant positions of conspecific calls when other factors such as pitch, duration and loudness are held constant. For example, Owren (1990a,b) used linear predictive coding to create synthetic versions of vervet monkey alarm calls in which vocal tract filtering could be controlled independently of the temporal envelope and source waveform (pitch). In a classification task, the judgments of trained monkeys were shown to be strongly influenced by the vocal tract function used to synthesize calls. Animals are also spontaneously sensitive to changes in formants of synthesized conspecific calls. Fitch and Kelley (2000) found that whooping cranes dishabituated when presented with synthetic contact calls in which formants frequencies are modified. Similarly findings have been reported for dishabituation towards formant shifted calls in red deer (Reby et al., 2005) and rhesus macaques (Fitch and Fritz, 2006). The results of these studies, in which the acoustic features of calls are precisely controlled, emphasize that timbre perception can be defined in animals as in humans; as the quality that distinguishes sounds of equal pitch, loudness or duration.

It is worth noting that timbre in animal communication may result from sources other than vocal tract filtering. For example when compared to mammals and birds, anuran species such as frogs and toads have relatively simple supra-laryngeal structures that provide a limited opportunity for resonant filtering. Nonetheless, several species of frogs produce vocalizations with spectral prominences resembling formants. Experiments in heliox environments have demonstrated that these prominences are not the result of cavity resonance in the vocal tract but rather are likely to be introduced at the sound source (Rand and Dudley, 1993). Resonant filtering opportunities may also be limited in small animals such as mice pups that can produce calls with formant-like spectral prominences at low frequencies (Ehret and Riecke, 2002; Geissler and Ehret, 2002). These low-frequency spectral prominences are unlikely to result from vocal tract filtering as the vocal tracts of pups are too short (Fitch and Fritz, 2006). Instead, such prominences are likely to be introduced at the sound source within the larynx (Roberts, 1975; Fitch and Fritz, 2006). Low frequency spectral prominences of mouse pup and anuran calls may thus provide examples of laryngeal (rather than supra-laryngeal) timbre and, in the case of the mouse, there is evidence that the positions of such prominences influence behavior (Ehret and Riecke, 2002).

Timbre takes on an additional ecological significance when introduced through vocal tract filtering. This is because the frequencies of formants introduced by the vocal tract are dependent on vocal tract length: As the vocal tract becomes longer, formants become lower in frequency and less dispersed (Fitch, 1997; Riede and Fitch, 1999; Reby and Mccomb, 2003; Rendall et al., 2005; Harris et al., 2006; Sanvito et al., 2007; Vannoni and Mcelligott, 2008). This makes it possible for listeners to infer the size of callers from the timbre of vocalizations containing formants (Fitch and Giedd, 1999; Reby et al., 2005; Smith et al., 2005; Charlton et al., 2007; Ghazanfar et al., 2007). The ability to determine size from formants may be helpful in several regards: Firstly, size may be indicative of caller gender or identity (Rendall et al., 1996, 1998; Feinberg et al., 2005). Secondly, size is a critical factor that must be separated from call identity when normalizing across speakers to achieve perceptual constancy. Finally, as size often indicates fitness and competitive ability, vocalization timbre may play an important role in mate selection (Feinberg et al., 2005; Charlton et al., 2007, 2008) and territorial defense (Fitch, 1999). Thus in addition to information about identity of a call, timbre conveys biologically important signals about the caller themselves that could determine reproductive success. Timbre production in vocalizations is therefore likely to be the subject of intensive selection pressure (Fitch, 2000; Fitch and Reby, 2001).

## Neural correlates of timbre perception

### The neural locus of timbre sensitivity: peripheral encoding of timbre cues

As in the behavioral studies reviewed above, much investigation into the neural basis of sound timbre has focused on vowel sounds. Neural coding of vowels begins in the auditory nerve where auditory nerve fibers (ANFs) relay sound information from the cochlea to the central auditory system. Information about vowel sounds may be relayed using place (the activation pattern across ANFs tuned to different sound frequencies) and temporal representations (the temporal firing pattern of fibers). Temporal information in the discharge patterns of populations of ANFs provide a robust estimate of the frequency spectra of single vowels in the cat (Young and Sachs, 1979; Delgutte and Kiang, 1984a) and of concurrently presented vowel pairs in the guinea pig (Palmer, 1990). Furthermore, temporal information is preserved in the presence of background noise; in ANFs whose characteristic frequency (CF, the frequency to which a neuron is most sensitive) was close to the formant frequencies, noise did not affect the temporal representations of vowels that could be extracted from ANF responses whereas peaks in the discharge rate in response to vowel stimuli are nearly eliminated in the presence of masking noise (Delgutte and Kiang, 1984b). Natural vowel sounds are periodic, due to the way in which the vocal folds vibrate as air is forced over them. The resulting vibrations have a harmonic structure. This periodicity makes extracting temporal information straight-forwards. However, vowels can also be aperiodic when the vocal folds remain static, leading to whispered speech. Yet information about the timbre of a whispered vowel can also be extracted from the temporal properties of ANF discharge rates. Temporal-place representations thus provide an accurate reflection of the stimulus spectrum for both periodic and aperiodic vowel sounds (Voigt et al., 1982).

Therefore, at the level of the auditory nerve, the temporal and spectral characteristics that psychophysically determine the timbre of a sound source are represented in the population activity of ANFs. However in order to recognize the timbre of, for example, a violin the representation of sound-source acoustics present across ANFs must be transformed so that certain acoustic features, such as the spectral envelope, are represented in a manner that is invariant to other parameters, such as the fine temporal and spectral details that determine pitch. At higher levels it seems likely that single neurons or neural populations must be able to abstract or generalize across certain features in order to recognize or identify a sound source. This latter stage of processing is not the subject of this review (though see Bizley and Cohen, 2013).

### Auditory cortex and timbre discrimination

Where in the brain does the process occur of integrating information across frequency channels in order to extract spectral envelope cues? The representation of vowels in the ventral cochlear nucleus is not qualitatively different from that seen in ANFs, although this varies by neuronal subtype; primary-like units resemble ANF responses, while chopper units exhibit larger differences in firing rate for units with CFs at the peak versus the trough of a formant. Chopper units are also more robust to changes in sound level of vowels than primary like units or ANFs (May et al., 1996, 1998). Studies investigating vowel encoding at higher auditory centers have almost exclusively focused on the Auditory Cortex. Since frequency tuning is broader in auditory cortex there is greater potential to integrate across the range of frequencies necessary to represent formant relationships. Importantly, there is also evidence that an intact auditory cortex is key for timbre perception.

Observations of human patients and studies in animals with brain lesions suggest that an intact auditory cortex is required for timbre sensitivity and that, in particular, non-primary auditory cortex plays a key role. Observations of human stroke patients pinpoint auditory cortex as important for musical timbre discrimination, with a particular emphasis on the requirement for an intact right auditory cortex for spectral and temporal timbre discrimination (Milner and Mountcastle, 1962; Samson and Zatorre, 1994). More recent studies support the idea that the right auditory cortex may be specialized for timbre processing, but suggest that both left and right auditory cortex may be important. Patients with left temporal lobe lesions were shown to be unimpaired in discriminating single tones based on their onset properties (i.e., temporal based timbre cues), but when such tones were presented in the context of a melody these same patients were unable to perform dissimilarity judgments. Patients with right hemisphere lesions were impaired on both single tone and melodic comparisons (Samson et al., 2002). Lesion studies in rats have emphasized the contribution of higher auditory cortical areas over primary auditory cortex as only damage to the former impairs vowel discrimination (Kudoh and Shibuki, 2006). From these studies we can conclude that an intact auditory cortex is required for timbre discrimination. Nevertheless more detailed reversible inactivation studies of specific cortical fields in animals trained to perform timbre discrimination would provide interesting insights into the neural architecture underlying spectral, and in particular temporal, timbre perception.

Functional imaging studies in human subjects allow us to more precisely determine the neural networks that are activated during timbre processing. We will first consider the representation of vowel sounds, before considering how other timbre-related percepts are encoded in auditory cortex. Vowel sounds elicit activity that is consistent with processing occurring across a series of hierarchically organized areas (Rauschecker and Scott, 2009). Neural sensitivity to vowel class might result as a consequence of the underlying acoustic differences between vowels (see Section 1.1) or may result from language-specific processes. Sensitivity to vowel class emerges in higher areas such as the anterior Superior Temporal Cortex (STC; Obleser et al., 2006) and cortical maps of vowel space can be extracted from neural signals in a way that reflects acoustical differences (Scharinger et al., 2011), suggesting both factors are important. At the cellular level, a topographic representation of F2–F1 distance has been observed in the primary auditory cortex of gerbils using 2-deoxyglucose activation patterns (Ohl and Scheich, 1997) suggesting that neural representations of the acoustical features that differentiate vowel sounds are not uniquely human.

The timbre of someone’s voice is an important cue to his or her identity. It is possible to decode both vowel identity (“what”) and speaker identity (“who”) from patterns of activation observed across voxels using fMRI (Formisano et al., 2008). Signals from a wide and bilaterally distributed variety of regions in Superior Temporal Gyrus (STG) including the anterior-lateral Heschl’s Gyrus (HG), the Planum Temporale (PT) and extended portions of Superior Temporal Sulcus (STS) and STG contributed to decoding. Discriminative patterns for speaker identity were more restricted and right-lateralized than those for vowels but still included both primary and non-primary auditory areas; specifically lateral HG, Heschl’s Sulcus and three regions clustered along the anterior-posterior axis of the right STS which were interspersed with vowel sensitive regions (Formisano et al., 2008). This supports the idea that widely distributed networks of areas underlie timbre perception with both low-level (i.e., vowel identity) and high-level (speaker identity) stimulus features being encoded at multiple levels of the cortical hierarchy. Staeren et al. (2009) used acoustically matched stimuli (guitars, cats and singers) to explore category representation in auditory cortex. Since sounds across acoustic categories were matched in pitch, timbre was the key acoustic determinant of category. These authors found evidence that spatial patterns of activation differentiated the three acoustic categories in a range of higher auditory areas including antero-lateral HG, the PT, and the posterior STG and/or STS. Information about the pitch of these sounds was also broadly distributed across multiple cortical fields. More generally the spectral envelope of a sound conveys information about its acoustic scale as well as its identity (van Dinther and Patterson, 2006; Von Kriegstein et al., 2006). Correspondingly, spectral envelope activates STG bilaterally whether the changes in the stimulus relate to its identity or its size. However in the left posterior STG, neural responses are specific to acoustic scale in human voices while the anterior temporal lobe and intraparietal sulcus demonstrate sensitivity to changes in acoustic scale across voices, animal vocalizations and musical instruments (Von Kriegstein et al., 2007).

The observation that timbre sensitivity is distributed across multiple cortical fields might appear surprising, but it may be that different cortical fields exploit similar information for different functions. Deike et al. (2004) used fMRI to measure activity in human auditory cortex while listeners were presented with sequences of harmonic complex tones with alternating spectral envelopes, which were tailored to evoke organ-like and trumpet-like timbres. The results showed greater activation in the left but not in right auditory cortex during the presentation of sequences with alternating spectral envelopes (and thus perceived timbre), compared to the condition with a constant spectral envelope. The authors interpreted this result as evidence for a selective involvement of left auditory cortex during stream segregation based on timbre cues conveyed by spectral differences. Thus even though right auditory cortex seems likely to be specialized for processing the acoustic features that define timbre, other areas—notably the posterior fields in the left auditory cortex—may be specialized for the exploitation of such acoustic cues for specific functions.

Studies which combine imaging with computational techniques such as dynamic causal modeling (DCM) provide additional power in disentangling the complex network of activation that is elicited while subjects are performing a listening task, and enable theories of information processing to be tested. For example, Kumar et al. (2007) explored the representation of spectral envelope in auditory cortex. DCM suggested that processing was performed in serial from HG (primary auditory cortex) to the PT and then to the STS. While there has been some recent debate about the use of DCM (Lohmann et al., 2012; Breakspear, 2013; Friston et al., 2013; Lohmann et al., 2013) the findings of Kumar et al. were supported by previous work suggesting that a processing hierarchy within auditory cortex exists (Warren et al., 2005; Kumar et al., 2007). Kumar et al. (2007) speculated that object features were extracted in primary areas and that further processing took place within PT, where it was proposed a series of “spectral templates” exist, i.e., templates that enable the extraction of particular spectral features or envelopes. These templates would enable neural activity to show tuning to spectral envelope and some degree of invariance to fine temporal structure (Kumar et al., 2007). Whether such templates can be observed as a single-neuron receptive field, or are an emergent network property remains to be determined.

### How do single neurons in auditory cortex encode timbre?

What about the single neuron computations that underlie timbre perception? Are there, for example, neurons in auditory cortex whose response properties are compatible with the idea of spectral templates as outlined above? Perceptual constancy likely requires an invariant representation of spectral timbre, which in turn requires that a neuron integrates across frequencies in order to extract a representation of spectral envelope that is independent of the fine spectral details.

Neurons throughout ferret auditory cortex are sensitive to sound timbre (Bizley et al., 2009). However, when artificial vowel stimuli were varied simultaneously in pitch and location as well as timbre, neural responses both in core and early belt areas were sensitive to multiple sound features (Bizley et al., 2009). The authors found no evidence either for a cortical field specialized for timbre—or pitch or location—processing, or a subset of neurons whose responses were sensitive only to changes in vowel identity. While, on average, neurons in the primary fields Primary Auditory Cortex (A1) and Anterior Auditory Field (AAF) were more sensitive to timbre than those in other fields, there was no evidence for an invariant representation of vowel identity. While auditory cortical neurons differed in their sensitivity to pitch, location, and timbre, the responses of most neurons were determined to some degree by combinations of these perceptual features—for example, a neuron might show a preference for a particular vowel in the stimulus set and for high pitch sounds. The demonstration that neuron’s whose responses are sensitive to timbre, pitch and location are seen throughout auditory cortex is consistent with the observation that both high and low-level stimulus features are represented throughout auditory cortex in humans (Staeren et al., 2009). Nevertheless, when the ferret auditory cortex responses were analyzed in discrete time-bins there was evidence that information about sound features could be extracted independently at different periods in the neural response. For example, information about sound timbre occurred earlier in the neural response shortly after stimulus onset, whereas the sustained response carried information about the fundamental frequency of the sound (Walker et al., 2011). Response times in animals trained to detect changes with reaction times being significantly shorter when detecting changes in sound timbre (Walker et al., 2011). Neural responses in auditory core and early belt areas therefore seem to contain an “implicit” code for object identity (Bizley and Cohen, 2013). Whether this implicit representation is converted to one that explicitly represents timbre in a manner that is invariant to changes in other stimulus dimensions remains a fruitful avenue for further investigation. It may be that higher brain areas contain such a representation, or that such a representation only emerges under the appropriate behavioral constraints.

**Figure 3:**
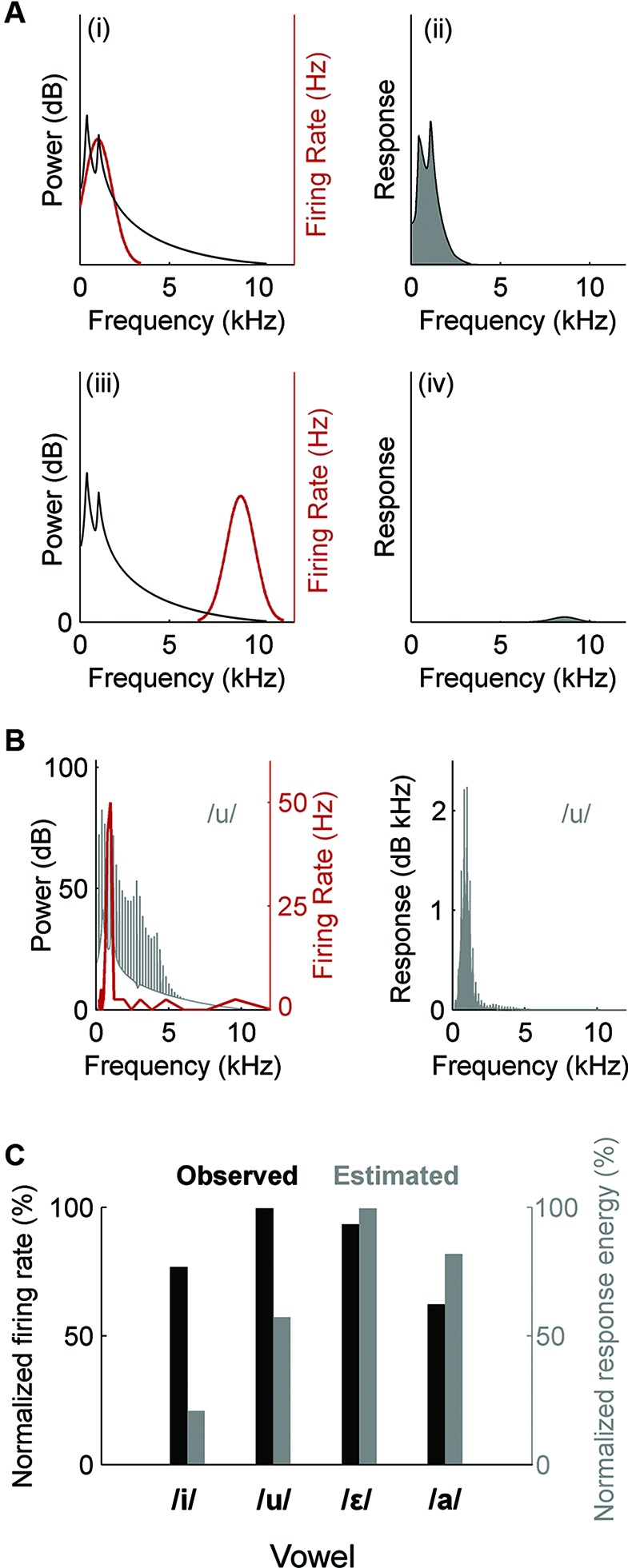
**Estimation of neural responses to vowels based on SRF.** The power spectrum of a vowel is multiplied by the SRF of a neuron to produce an estimated response spectrum. The area under the spectrum is taken as the response energy; a measure of the neurons response magnitude. **(A)** When the vowel spectrum (black) and SRF (red) overlap (i), the neuron’s response energy is predicted to be large (ii). In contrast, if the vowel spectrum and SRF are separated (iii), the neuron’s response is predicted to be small (iv). **(B)** Left: SRF (red) recorded from a multi-unit cluster within auditory cortex of an anesthetized ferret and the spectrum of the vowel /u/. Right: Estimated response energy of unit to /u/. **(C)** Comparison of the estimated (grey) and observed (black) responses of the unit in **(B)** to a series of vowels. Firing rate and response energy are normalized for comparison. Note that the pattern of vowel discrimination by firing rate differs from the pattern estimated from response energy. Observed responses were measured as the mean firing rate across 20 presentations of each vowel.

What determines whether a single neuron is sensitive to the timbre of a vowel sound? An open question is to what extent the timbre sensitivity that we observe in auditory cortex arises due to neurons integrating across frequencies in order to estimate the spectral envelope, or whether timbre sensitivity merely arises due to the frequency-specific properties of auditory cortical neuron receptive fields. In order to better understand how neural selectivity for a particular vowel timbre might occur, our ongoing work is exploring what stimulus features underlie timbre selectivity and to what extent we can predict neural sensitivity to timbre based on pure tone frequency tuning. Neural selectivity to a vowel sound might arise because formant peaks in some vowels, but not others, fall close to the neuron’s CF—depending on the precise location of formants more or less energy may fall close to a neuron’s CF and will drive the neuron to fire a greater (or lesser) number of spikes. Since the pure tone frequency tuning of auditory cortical neurons is typically broader than that observed at lower auditory centers we expanded this to consider the Spectral Receptive Field (SRF) estimated from the frequency response area (FRA), which is measured by presenting a range of tone frequencies across multiple intensities. If an auditory neuron acts as a linear filter then it should be possible to predict the relative ability of different vowel sounds to excite a given cell from the SRF (Figure 3A). Note that this method utilizes the whole spectrum of the vowel (rather than just the location of the formant peaks) and that using the SRF enables us to take into account the full frequency tuning function (at a single sound level) and therefore captures features such as multi-peaked frequency tuning observed at the single neuron level (e.g., Sutter and Schreiner, 1991; Kadia and Wang, 2003; Bizley et al., 2005). However, if the neuron integrates across frequencies in some non-linear way—perhaps because its receptive field also includes regions of inhibition that are only visible by performing two-tone suppression experiments, or mapping spectrotemporal receptive fields (STRF) with sounds such as random chords or dynamic ripples—then the frequency tuning of a cell, as defined by the FRA, will be a poor predictor of the cell’s vowel sensitivity. It has been demonstrated that at and between the midbrain Inferior Colliculus (IC) and auditory cortex the neural encoding of both the spectral shape (identity) and spectral scale (an indicator of vocal tract length) of vowel sounds becomes substantially more non-linear than at earlier processing stations, where tuning properties can be well predicted from a linear model of the FRA (Schebesch et al., 2010). Very few neurons in the midbrain and auditory cortex showed a significant shape or scale preference suggesting that this linear model was a poor predictor. Recordings in our lab made throughout primary and non-primary auditory cortex in ferrets also suggest that frequency tuning as derived from the FRA is an imperfect predictor of the tuning of a neuron to particular timbres (Figures 3B, C). However, linear estimates of the STRFs of A1 neurons made using broadband ripple stimuli enabled the relative responses to different vowels to be well predicted for a majority (71%) of A1 neurons (Versnel and Shamma, 1998). This suggests that aspects of neuronal sensitivity characterized by STRFs but not FRAs—such as temporal dynamics of sensitivity and the occurrence of inhibition—make important contributions in auditory cortical responses to vowels.

Isolated steady-state vowel sounds have very simple temporal structures (seen for both spoken and artificially generated vowels, in Figures 1A, B). However natural sounds and notably musical instruments vary in their temporal as well as spectral content. Computational and behavioral approaches have highlighted the importance of the joint temporal and spectral features in musical instrument identification (Samson and Zatorre, 1994; McAdams et al., 1995; Samson et al., 2002; Patil et al., 2012; Elliott et al., 2013). Modelling studies demonstrate that an array of primary auditory cortical neurons contains the necessary response complexity to provide the multi-dimensional stimulus mapping necessary for musical timbre discrimination: Neural tuning can vary along at least three dimensions; CF, spectral shape which can be very broad or very narrow, and temporal dynamics which range from slow to fast (Patil et al., 2012). Models incorporating these tuning features, based on linear descriptors of auditory neuron responses are capable of accurately discriminating the identity of a musical instrument across different pitches and playing styles. However best performance was only observed when a non-linear decision boundary was used, suggesting that a linear spectrotemporal analysis must be accompanied by nonlinearities such as divisive normalization or synaptic depression.

How should we relate these insights into single neuron timbre-sensitivity to the activation patterns observed using functional imaging methods? Based on the human imaging work discussed at the beginning of this section we might expect to see that neurons sensitive to timbre are found in a particular cortical field. Yet, the available electrophysiological data suggests that neurons throughout auditory cortex are sensitive to sound timbre. However, it is important to note the methodological constraints of these two different methods; fMRI methods often rely on subtracting activation patterns from two different stimulus classes—for example the activity when spectral envelope changes versus when fine spectral detail changes—to highlight timbre sensitivity. Analysis methods that use multivariate pattern recognition algorithms to decode distinct patterns of activation (e.g., Formisano et al., 2008) offer an alternative, although one that is still notably different from the analysis of spiking data which looks not at whether neurons are active or not, but rather whether they are tuned to a particular stimulus dimension. Given the fundamental difference in these approaches it is not surprising that they offer what sometimes appear to be contrasting insights into the underlying neural mechanisms. Studies employing fMRI methods or optical imaging in animals might provide a link between BOLD signals and single neuron neurophysiology ultimately allowing us to better integrate human and animal work. Reconciling invariant timbre perception with distributed and non-invariant neural sensitivity requires employing different experimental approaches, as outlined below.

## Summary and future outlook

Timbre is a complex perceptual property of sound that results from multiple acoustic cues and plays a critical role in our perception of music and speech. The ability to perceive timbre is not limited to humans but is shared by many animal species; most likely because of its additional significance in non-human vocal communication. Timbre perception by animals provides opportunities to understand the broader ecological importance of timbre and to study underlying neural mechanisms. So far, single cell recordings in animal models and functional imaging in humans have demonstrated that neural sensitivity to sound timbre is distributed widely across auditory cortex. However, we still have a limited understanding of how spectral timbre is extracted by the brain. Neurophysiological investigations have yet to tackle the multidimensional aspects of timbre perception. In addition to urging the expansion of studies to include temporal and spectrotemporal aspects of timbre perception, we argue that two experimental approaches are key to furthering our understanding of the neural basis of timbre discrimination. Firstly, recordings in animals that are actively discriminating sound timbre may provide insights into how timbre is extracted independently of other features such as pitch, loudness or location in space. Ideally such recordings should enable multiple neurons to be recorded simultaneously since it might be large neuronal populations rather than small subsets of neurons that invariantly and unambiguously represent multiple sound features. Modeling studies (e.g., Patil et al., 2012) provide predictions about how auditory cortical activity might be decoded in order to support timbre perception. Secondly, a focus on the underlying computations that result in a representation of timbre might prove beneficial. Warren et al. (2005) proposed that an area in the right STS of humans was responsible for a particular computational step—namely spectral envelope extraction. Searching for and exploring single neuron correlates of such a computation, rather than sensitivity to a particular subset of sounds, would enable a mechanistic understanding of how timbre might be extracted.

## Conflict of interest statement

The authors declare that the research was conducted in the absence of any commercial or financial relationships that could be construed as a potential conflict of interest.

## References

[B1] Andrew R. J. (1976). Use of formants in the grunts of baboons and other nonhuman primates. Ann. N Y Acad. Sci. 280, 673–693 10.1111/j.1749-6632.1976.tb25530.x 827958

[B2] Assmann P. F. Katz W. F. (2005). Synthesis fidelity and time-varying spectral change in vowels. J. Acoust. Soc. Am. 117, 886–895 10.1121/1.1852549 15759708

[B3] Assmann P. F. K. Katz W. F. (2000). Time-varying spectral change in the vowels of children and adults. J. Acoust. Soc. Am. 108, 1856–1866 10.1121/1.1289363 11051512

[B4] Baru A. V. (1975). “Discrimination of synthesized vowels [a] and [i] with varying parameters (fundamental frequency, intensity, duration and number of formants) in dog,” in Auditory Analysis and Perception of Speech, eds Fant G. Tatham M. A. A. (New York: Academic), 173–191

[B5] Beddor P. S. Hawkins S. (1990). The influence of spectral prominence on perceived vowel quality. J. Acoust. Soc. Am. 87, 2684–2704 10.1121/1.399060 2373803

[B6] Behar I. Cronholm J. N. Loeb M. (1965). Auditory sensitivity of the rhesus monkey. J. Comp. Physiol. Psychol. 59, 426–428 10.1037/h0022047 14313786

[B7] Berger K. W. (1964). Some factors in the recognition of timbre. J. Acoust. Soc. Am. 36, 1888–1891 10.1121/1.1919287

[B8] Bizley J. K. Cohen Y. E. (2013). The what, where and how of auditory object processing. Nat. Rev. Neurosci. 14, 693–707 10.1038/nrn3565 24052177 PMC4082027

[B9] Bizley J. K. Nodal F. R. Nelken I. King A. J. (2005). Functional organization of ferret auditory cortex. Cereb. Cortex 15, 1637–1653 10.1093/cercor/bhi042 15703254

[B10] Bizley J. K. Walker K. M. M. King A. J. Schnupp J. W. H. (2013). Spectral timbre perception in ferrets: discrimination of artificial vowels under different listening conditions. J. Acoust. Soc. Am. 133, 365–376 10.1121/1.4768798 23297909 PMC3783993

[B11] Bizley J. K. Walker K. M. Silvermann B. W. King A. J. Schnupp J. W. (2009). Interdependent encoding of pitch, timbre and spatial location in auditory cortex. J. Neurosci. 29, 2064–2075 10.1523/jneurosci.4755-08.2009 19228960 PMC2663390

[B12] Bladon A. (1983). Two-formant models of vowel perception: shortcomings and enhancements. Speech Commun. 2, 305–313 10.1016/0167-6393(83)90047-x

[B13] Breakspear M. (2013). Dynamic and stochastic models of neuroimaging data: a comment on Lohmann et al. Neuroimage 75, 270–274; discussion 279–281 10.1016/j.neuroimage.2012.02.047 22387473

[B14] Burdick C. K. Miller J. D. (1975). Speech perception by the chinchilla: discrimination of sustained /a/ and /i/. J. Acoust. Soc. Am. 58, 415–427 10.1121/1.380686 1184835

[B15] Burgoyne J. A. McAdams S. (2008). “A meta-analysis of timbre perception using nonlinear extensions to CLASCAL,” in Computer Music Modeling and Retrieval. Sense of Sounds: 4th International Symposium, CMMR 2007, Copenhagen, Denmark, August 27–31, 2007. Revised Papers, eds Kronland-Martinet R. Ystad S. Jensen K. (Berlin: Springer-Verlag), 181–202

[B16] Caclin A. McAdams S. Smith B. K. Winsberg S. (2005). Acoustic correlates of timbre space dimensions: a confirmatory study using synthetic tones. J. Acoust. Soc. Am. 118, 471–482 10.1121/1.1929229 16119366

[B17] Campbell M. Greated C. (1994). The Musician’s Guide to Acoustics. (Oxford: OUP).

[B18] Carlson R. Granstrom B. Klatt D. (1979). Vowel perception: the relative perceptual salience of selected acoustic manipulations. STL-QPSR 20, 73–83

[B19] Chaigne A. Lambourg C. (2001). Time-domain simulation of damped impacted plates. I. Theory and experiments. J. Acoust. Soc. Am. 109, 1433–1447 10.1121/1.1354200 11325114

[B20] Charlton B. D. Ellis W. A. H. Mckinnon A. J. Cowin G. J. Brumm J. Nilsson K. (2011). Cues to body size in the formant spacing of male koala (Phascolarctos cinereus) bellows: honesty in an exaggerated trait. J. Exp. Biol. 214, 3414–3422 10.1242/jeb.061358 21957105

[B21] Charlton B. D. Reby D. Mccomb K. (2007). Female red deer prefer the roars of larger males. Biol. Lett. 3, 382–385 10.1098/rsbl.2007.0244 17550876 PMC2390678

[B22] Charlton B. D. Reby D. Mccomb K. (2008). Effect of combined source (F0) and filter (formant) variation on red deer hind responses to male roars. J. Acoust. Soc. Am. 123, 2936–2943 10.1121/1.2896758 18529210

[B23] Charlton B. D. Zhihe Z. Snyder R. J. (2009). The information content of giant panda, Ailuropoda melanoleuca, bleats: acoustic cues to sex, age and size. Anim. Behav. 78, 893–898 10.1016/j.anbehav.2009.06.029

[B24] Christovich L. A. Lublinskaya V. V. (1979). The ‘center of gravity’ effect in vowel spectra and critical distance between the formants: psychoacoustical study of the perception of vowel-like stimuli. Hear. Res. 1, 185–195 10.1016/0378-5955(79)90012-1

[B25] Coleman M. N. (2009). What do primates hear? A meta-analysis of all known nonhuman primate behavioral audiograms. Int. J. Primatol. 30, 55–91 10.1007/s10764-008-9330-1

[B26] Cynx J. Williams H. Nottebohm F. (1990). Timbre discrimination in Zebra Finch (Taeniopygia guttata) song syllables. J. Comp. Psychol. 104, 303–308 10.1037//0735-7036.104.4.303 2282781

[B27] Deike S. Gaschler-Markefski B. Brechmann A. Scheich H. (2004). Auditory stream segregation relying on timbre involves left auditory cortex. Neuroreport 15, 1511–1514 10.1097/01.wnr.0000132919.12990.34 15194885

[B28] Delattre P. Liberman A. M. Cooper F. S. Gerstman L. J. (1952). An experimental study of the acoustic determinants of vowel color; observations on one- and two-formant vowels synthesized from spectrographic patterns. Word 8, 195–210

[B29] Delgutte B. Kiang N. Y. (1984a). Speech coding in the auditory nerve: I. Vowel-like sounds. J. Acoust. Soc. Am. 75, 866–878 10.1121/1.390596 6707316

[B30] Delgutte B. Kiang N. Y. (1984b). Speech coding in the auditory nerve: V. Vowels in background noise. J. Acoust. Soc. Am. 75, 908–918 10.1121/1.390537 6707320

[B31] Dewson J. H. (1964). Speech sound discrimination by cats. Science 144, 555–556 10.1126/science.144.3618.555 14194105

[B32] Dewson J. H. (1968). Efferent olivocochlear bundle: some relationships to stimulus discrimination in noise. J. Neurophysiol. 31, 122–130 4966613 10.1152/jn.1968.31.1.122

[B33] Dewson J. H. Pribram K. H. Lynch J. C. (1969). Effects of ablations of temporal cortex upon speech sound discrimination in the monkey. Exp. Neurol. 24, 579–591 10.1016/0014-4886(69)90159-9 4979090

[B34] Dooling R. J. Brown S. D. (1990). Speech perception by budgerigars (Melopsittacus undulatus): spoken vowels. Percept. Psychophys. 47, 568–574 2367177 10.3758/bf03203109

[B35] Dubno J. R. Dorman M. F. (1987). Effects of spectral flattening on vowel identification. J. Acoust. Soc. Am. 82, 1503–1511 3693692 10.1121/1.395194

[B36] Ehret G. Riecke S. (2002). Mice and humans perceive multiharmonic communication sounds in the same way. Proc. Natl. Acad. Sci. U S A 99, 479–482 10.1073/pnas.012361999 11756654 PMC117585

[B37] Elliott T. M. Hamilton L. S. Theunissen F. E. (2013). Acoustic structure of the five perceptual dimensions of timbre in orchestral instrument tones. J. Acoust. Soc. Am. 133, 389–404 10.1121/1.4770244 23297911 PMC3548835

[B38] Eriksson J. L. Villa A. E. (2006). Learning of auditory equivalence classes for vowels by rats. Behav. Processes 73, 348–359 10.1016/j.beproc.2006.08.005 16997507

[B39] Feinberg D. R. Jones B. C. Little A. C. Burt D. M. Perrett D. I. (2005). Manipulations of fundamental and formant frequencies influence the attractiveness of human male voices. Anim. Behav. 69, 561–568 10.1016/j.anbehav.2004.06.012

[B40] Fitch W. T. (1997). Vocal tract length and formant frequency dispersion correlate with body size in rhesus macaques. J. Acoust. Soc. Am. 102, 1213–1222 10.1121/1.419022 9265764

[B41] Fitch W. T. (1999). Acoustic exaggeration of size in birds via tracheal elongation: comparative and theoretical analysis. J. Zool. London 248, 31–48 10.1017/s095283699900504x

[B42] Fitch W. T. (2000). The evolution of speech: a comparative review. Trends Cogn. Sci. 4, 258–267 10.1016/s1364-6613(00)01494-7 10859570

[B43] Fitch W. T. Fritz J. B. (2006). Rhesus macaques spontaneously perceive formants in conspecifics vocalizations. J. Acoust. Soc. Am. 120, 2132–2141 10.1121/1.2258499 17069311

[B44] Fitch W. T. Giedd J. (1999). Morphology and development of the human vocal tract: a study using magnetic resonance imaging. J. Acoust. Soc. Am. 106, 1511–1522 10.1121/1.427148 10489707

[B45] Fitch W. T. Kelley J. P. (2000). Perception of vocal tract resonances by Whooping Cranes Grus americana. Ethology 106, 559–574 10.1046/j.1439-0310.2000.00572.x

[B46] Fitch W. T. Reby D. (2001). The descended larynx is not uniquely human. Proc. Biol. Sci. 268, 1669–1675 10.1098/rspb.2001.1704 11506679 PMC1088793

[B47] Formisano E. De Martino F. Bonte M. Goebel R. (2008). “Who” is saying “what”? Brain-based decoding of human voice and speech. Science 322, 970–973 10.1126/science.1164318 18988858

[B48] Friston K. Daunizeau J. Stephan K. E. (2013). Model selection and gobbledygook: response to Lohmann et al. Neuroimage 75, 275–278; discussion 279–281 10.1016/j.neuroimage.2011.11.064 22155029

[B49] Geissler D. B. Ehret G. (2002). Time-critical integration of formants for perception of communication calls in mice. Proc. Natl. Acad. Sci. U S A 99, 9021–9025 10.1073/pnas.122606499 12070345 PMC124416

[B50] Ghazanfar A. A. Turesson H. K. Maier J. X. Van Dinther R. Patterson R. D. Logothetis N. K. (2007). Vocal tract resonances as indexical cues in rhesus monkeys. Curr. Biol. 17, 425–430 10.1016/j.cub.2007.01.029 17320389 PMC2361420

[B51] Giordano B. L. McAdams S. (2006). Material identification of real impact sounds: effects of size variation in steel, glass, wood, and plexiglass plates. J. Acoust. Soc. Am. 119, 1171–1181 10.1121/1.2149839 16521778

[B52] Grey J. M. (1977). Multidimensional perceptual scaling of musical timbres. J. Acoust. Soc. Am. 61, 1270–1277 10.1121/1.381428 560400

[B53] Grey J. M. Gordon J. W. (1978). Perceptual effects of spectral modifications on musical timbres. J. Acoust. Soc. Am. 63, 1493–1500 10.1121/1.381843

[B54] Harris T. R. Fitch W. T. Goldstein L. M. Fashing P. J. (2006). Black and white Colobus monkey (Colobus guereza) roars as a source of both honest and exaggerated information about body mass. Ethology 112, 911–920 10.1111/j.1439-0310.2006.01247.x

[B55] Harris K. S. Hoffmann H. S. Liberman A. S. Delattre P. C. Cooper F. S. (1958). Effect of third-formant transitions on the perception of the voiced stop consonants. J. Acoust. Soc. Am. 30, 122–126 10.1121/1.1909501

[B56] Hienz R. D. Aleszczyk C. M. May B. J. (1996). Vowel discrimination in cats: acquisition, effects of stimulus level, and performance in noise. J. Acoust. Soc. Am. 99, 3656–3668 10.1121/1.414980 8655797

[B57] Hienz R. D. Brady J. V. (1988). The acquisition of vowel discriminations by nonhuman primates. J. Acoust. Soc. Am. 84, 186–194 10.1121/1.396963 3411047

[B58] Hienz R. D. Jones A. M. Weerts E. M. (2004). The discrimination of baboon grunt calls and human vowel sounds by babboons. J. Acoust. Soc. Am. 116, 1692–1697 10.1121/1.1778902 15478436

[B59] Hienz R. D. Sachs M. B. Aleszczyk C. M. (1993). Frequency discrimination in noise: comparison of cat performances with auditory-nerve models. J. Acoust. Soc. Am. 93, 462–469 10.1121/1.405626 8423262

[B60] Hienz R. D. Sachs M. B. Sinnott J. M. (1981). Discrimination of steady-state vowels by blackbirds and pigeons. J. Acoust. Soc. Am. 70, 699–706 10.1121/1.386933

[B61] Hienz R. D. Stiles P. May B. J. (1998). Effects of bilateral olivocochlear lesions on vowel formant discrimination in cats. Hear. Res. 116, 10–20 10.1016/s0378-5955(97)00197-4 9508024

[B62] Hillenbrand J. Getty L. A. Clark M. J. Wheeler K. (1995). Acoustic characteristics of American English vowels. J. Acoust. Soc. Am. 97, 3099–3111 10.1121/1.411872 7759650

[B63] Hillenbrand J. M. Nearey T. M. (1999). Identification of resynthesized /hVd/ utterances: effects of formant contour. J. Acoust. Soc. Am. 105, 3509–3523 10.1121/1.424676 10380673

[B64] ISO:226 (2003). Normal Equal-Loudness Level Contours. (Geneva: International Organization for Standardization).

[B65] Ito M. Tsuchida J. Yano M. (2001). On the effectiveness of whole spectral shape for vowel perception. J. Acoust. Soc. Am. 110, 1141–1149 10.1121/1.1384908 11519581

[B66] Iverson P. Krumhansl C. L. (1993). Isolating the dynamic attributes of musical timbre. J. Acoust. Soc. Am. 94, 2595–2603 10.1121/1.407371 8270737

[B67] Kadia S. C. Wang X. (2003). Spectral integration in A1 of awake primates: neurons with single- and multipeaked tuning characteristics. J. Neurophysiol. 89, 1603–1622 10.1152/jn.00271.2001 12626629

[B68] Kasturi K. Loizou P. C. Dorman M. Spahr T. (2002). The intelligibility of speech with “holes” in the spectrum. J. Acoust. Soc. Am. 112, 1102–1111 10.1121/1.1498855 12243158

[B69] Kelly J. B. Kavanagh G. L. Dalton J. C. (1986). Hearing in the ferret (Mustela putorius): thresholds for pure tone detection. Hear. Res. 24, 269–275 10.1016/0378-5955(86)90025-0 3793642

[B70] Kewley-Port D. Watson C. S. (1994). Formant-frequency discrimination for isolated English vowels. J. Acoust. Soc. Am. 95, 485–496 10.1121/1.410024 8120259

[B71] Klatt D. H. (1982). “Prediction of perceived phonetic distance from critical-band spectra: a first step,” in Acoustics, Speech, and Signal Processing, IEEE International Conference on ICASSP ’82. : IEEE), 1278–1281

[B72] Klatt D. H. Stefanski R. A. (1974). How does a mynah bird imitate human speech? J. Acoust. Soc. Am. 55, 822–832 10.1121/1.1914607 4833078

[B73] Kojima S. (1990). Comparison of auditory functions in the chimpanzee and human. Folia Primatol. (Basel) 55, 62–72 10.1159/000156501 2227723

[B74] Kojima S. Kiritani S. (1989). Vocal-auditory functions in the Chimpanzee: vowel perception. Int. J. Primatol. 10, 199–213 10.1007/bf02735200

[B75] Kudoh M. Shibuki K. (2006). Sound sequence discrimination learning motivated by reward requires dopaminergic D2 receptor activation in the rat auditory cortex. Learn. Mem. 13, 690–698 10.1101/lm.390506 17142301 PMC1783622

[B76] Kuhl P. K. Miller J. D. (1975). Speech perception by the chinchilla: voiced-voiceless distinction in alveolar plosive consonants. Science 190, 69–72 10.1126/science.1166301 1166301

[B77] Kuhl P. K. Miller J. D. (1978). Speech perception by the chinchilla: identification functions for synthetic VOT stimuli. J. Acoust. Soc. Am. 63, 905–917 10.1121/1.381770 670558

[B78] Kumar S. Stephan K. E. Warren J. D. Friston K. J. Griffiths T. D. (2007). Hierarchical processing of auditory objects in humans. PLoS Comput. Biol. 3:e100 10.1371/journal.pcbi.0030100.eor 17542641 PMC1885275

[B79] Kunkler-Peck A. J. Turvey M. T. (2000). Hearing shape. J. Exp. Psychol. Hum. Percept. Perform. 26, 279–294 10.1037/0096-1523.26.1.279 10696618

[B80] Lakatos S. (2000). A common perceptual space for harmonic and percussive timbres. Percept. Psychophys. 62, 1426–1439 10.3758/bf03212144 11143454

[B81] Lakatos S. McAdams S. Causse R. (1997). The representation of auditory source characteristics: simple geometric form. Percept. Psychophys. 59, 1180–1190 10.3758/bf03214206 9401453

[B82] Li F. Menon A. Allen J. B. (2010). A psychoacoustic method to find the perceptual cues of stop consonants in natural speech. J. Acoust. Soc. Am. 127, 2599–2610 10.1121/1.3295689 20370041 PMC2865708

[B83] Liberman A. M. Cooper F. S. Shankweiler D. P. Studdert-Kennedy M. (1967). Perception of the speech code. Psychol. Rev. 74, 431–461 10.1037/h0020279 4170865

[B84] Liberman A. M. Delattre P. C. Cooper F. S. Gerstman L. J. (1954). The role of consonant-vowel transitions in the perception of the stop and nasal consonants. Psychol. Monogr. 68, 1–13 10.1037/h0093673

[B85] Lisker L. (1986). “Voicing” in English: A catalogue of acoustic features signalling /b/ versus /p/ in trochees. Lang. Speech. 29, 3–11 3657346 10.1177/002383098602900102

[B86] Lohmann G. Erfurth K. Muller K. Turner R. (2012). Critical comments on dynamic causal modelling. Neuroimage 59, 2322–2329 10.1016/j.neuroimage.2011.09.025 22001162

[B87] Lohmann G. Muller K. Turner R. (2013). Response to commentaries on our paper: critical comments on dynamic causal modelling. Neuroimage 75, 279–281 10.1016/j.neuroimage.2012.07.041 29768908

[B88] Lufti R. A. (2001). Auditory detection of hollowness. J. Acoust. Soc. Am. 110, 1010–1019 10.1121/1.1385903 11519569

[B89] Lufti R. A. (2007). “Human sound source identification,” in Auditory Perception of Sound Sources, eds Yost W. A. Popper A. N. Fay R. R. (New York: Springer), 13–42

[B90] May B. J. (2003). Physiological and psychophysical assessments of the dynamic range of vowel representations in the auditory periphery. Speech Commun. 41, 49–57 10.1016/s0167-6393(02)00092-4

[B156] May B. J. Huang A. L. E. Prell G. Hienz R. D. (1996). Vowel formant frequency discrimination in cats: comparison of auditory nerve representations and psychophysical thresholds. Aud. Neurosci. 3, 135–162 23599660 PMC3627498

[B155] May B. J. Prell G. S. Sachs M. B. (1998). Vowel representations in the ventral cochlear nucleus of the cat: effects of level, background noise, and behavioral state. J. Neurophysiol. 79, 1755–1767 9535945 10.1152/jn.1998.79.4.1755

[B91] McAdams S. (1999). Perspectives on the contribution of timbre to musical structure. Computer Music J. 23, 85–102 10.1162/014892699559797

[B92] McAdams S. Chaigne A. Roussarie V. (2004). The psychomechanics of simulated sound sources: material properties of impacted bars. J. Acoust. Soc. Am. 115, 1306–1320 10.1121/1.1645855 15058353

[B93] McAdams S. Giordano B. L. (2009). “The perception of musical timbre,” in Oxford Handbook of Music Psychology, eds Hallam S. Cross I. Thaut M. (New York: OUP), 72–80

[B94] McAdams S. Roussarie V. Chaigne A. Giordano B. L. (2010). The psychomechanics of simulated sound sources: material properties of impacted thin plates. J. Acoust. Soc. Am. 128, 1401–1413 10.1121/1.3466867 20815474

[B95] McAdams S. Winsberg S. Donnadieu S. De Soete G. Krimphoff J. (1995). Perceptual scaling of synthesized musical timbres: common dimensions,specificities, and latent subject classes. Psychol. Res. 58, 177–192 10.1007/bf00419633 8570786

[B96] Miller J. R. Carterette E. C. (1975). Perceptual space for musical structures. J. Acoust. Soc. Am. 58, 711–720 10.1121/1.380719 1184844

[B97] Milner B. (1962). “Laterality rffects in audition,” in Interhemispheric Relations and Cerebral Dominance, eds Mountcastle V. (Baltimore: Johns Hopkins Press), 177–192

[B98] Molis M. R. (2005). Evaluating models of vowel perception. J. Acoust. Soc. Am. 118, 1062–1071 10.1121/1.1943907 16158661

[B99] Nowicki S. (1987). Vocal tract resonances in oscine bird sound production: evidence from birdsongs in a helium atmosphere. Nature 325, 53–55 10.1038/325053a0 3796738

[B100] Obleser J. Boecker H. Drzezga A. Haslinger B. Hennenlotter A. Roettinger M. (2006). Vowel sound extraction in anterior superior temporal cortex. Hum. Brain Mapp. 27, 562–571 10.1002/hbm.20201 16281283 PMC6871493

[B101] Ohl F. W. Scheich H. (1997). Orderly cortical representation of vowels based on formant interaction. Proc. Natl. Acad. Sci. U S A 94, 9440–9444 10.1073/pnas.94.17.9440 9256501 PMC23209

[B102] Ohms V. R. Escudero P. Lammers K. Ten Cate C. (2012). Zebra finches and Dutch adults exhibit the same cue weighting. Anim. Cogn. 15, 155–161 10.1007/s10071-011-0441-2 21761144 PMC3281197

[B103] Ohms V. R. Gill A. Van Heijningen C. A. Beckers G. J. Ten Cate C. (2010). Zebra finches exhibit speaker-independent phonetic perception of human speech. Proc. Biol. Sci. 277, 1003–1009 10.1098/rspb.2009.1788 19955157 PMC2842761

[B104] Okanoya K. Dooling R. J. (1987). Hearing in Passerine and Psittacine birds: a comparative study of absolute and masked auditory thresholds. J. Comp. Psychol. 101, 7–15 10.1037//0735-7036.101.1.7 3568610

[B105] Owren M. J. (1990a). Acoustic classification of alarm calls by Vervet monkeys (Cercopithecus aethiops) and Humans (Homo sapiens): I. Natural calls. J. Comp. Psychol. 104, 20–28 10.1037//0735-7036.104.1.20 2354626

[B106] Owren M. J. (1990b). Acoustic classification of alarm calls by vervet monkeys (Cercopithecus aethiops) and humans (Homo sapiens): II. Synthetic calls. J. Comp. Psychol. 104, 29–40 10.1037//0735-7036.104.1.29 2354627

[B107] Palmer A. R. (1990). The representation of the spectra and fundamental frequencies of steady-state single- and double-vowel sounds in the temporal discharge patterns of guinea pig cochlear-nerve fibers. J. Acoust. Soc. Am. 88, 1412–1426 10.1121/1.400329 2229676

[B108] Patil K. Pressnitzer D. Shamma S. Elhilali M. (2012). Music in our ears: the biological bases of musical timbre perception. PLoS Comput. Biol. 8:e1002759 10.1371/journal.pcbi.1002759 23133363 PMC3486808

[B109] Peterson G. E. Barney H. L. (1952). Control methods used in a study of vowels. J. Acoust. Soc. Am. 24, 175–184 10.1121/1.1906875

[B110] Pickett J. M. (1957). Perception of vowels heard in noises of various spectra. J. Acoust. Soc. Am. 29, 613–620 10.1121/1.1908983

[B111] Plomp R. Pols L. C. W. van der Geer J. P. (1967). Dimensional analysis of vowel spectra. J. Acoust. Soc. Am. 41, 707–712 10.1121/1.1910398

[B112] Plomp R. Steeneken H. J. M. (1969). Effect of phase on the timbre of complex tones. J. Acoust. Soc. Am. 46, 409–421 10.1121/1.1911705 5804112

[B113] Pols L. C. W. Van Der Kamp L. J. T. Plomp R. (1969). Perceptual and physical space of vowel sounds. J. Acoust. Soc. Am. 46, 458–467 10.1121/1.1911711 5804118

[B114] Potter R. K. Steinberg J. C. (1950). Towards the specification of speech. J. Acoust. Soc. Am. 22, 807–820 10.1121/1.1906694

[B115] Rand A. S. Dudley R. (1993). Frogs in helium: the anuran vocal sac is not a cavity resonator. Physiol. Zool. 66, 793–806

[B116] Rauschecker J. P. Scott S. K. (2009). Maps and streams in the auditory cortex: nonhuman primates illuminate human speech processing. Nat. Neurosci. 12, 718–724 10.1038/nn.2331 19471271 PMC2846110

[B117] Reby D. Mccomb K. (2003). Anatomical constraints generate honesty: acoustic cues to age and weight in the roars of red deer stags. Anim. Behav. 65, 519–530 10.1006/anbe.2003.2078

[B118] Reby D. Mccomb K. Cargnelutti B. Darwin C. Fitch W. T. Clutton-Brock T. (2005). Red deer stags use formants as assessment cues during intrasexual agonistic interactions. Proc. Biol. Sci. 272, 941–947 10.1098/rspb.2004.2954 16024350 PMC1564087

[B119] Rendall D. Kollias S. Ney C. (2005). Pitch (F0) and formant profiles of human vowels and vowel-like baboon grunts: the role of vocalizer body size and voice-acoustic allometry. J. Acoust. Soc. Am. 117, 944–955 10.1121/1.1848011 15759713

[B120] Rendall D. Owren M. J. Rodman P. S. (1998). The role of vocal tract filtering in identity cueing in rhesus monkey (Macaca mulatta) vocalizations. J. Acoust. Soc. Am. 103, 602–614 10.1121/1.421104 9440345

[B121] Rendall D. Rodman P. S. Emond R. E. (1996). Vocal recognition of individuals and kin in free-ranging rhesus monkeys. Anim. Behav. 51, 1007–1015 10.1006/anbe.1996.0103

[B122] Riede T. Fitch T. (1999). Vocal tract length and acoustics of vocalization in the domestic dog (Canis familiaris). J. Exp. Biol. 202, 2859–2867 10504322 10.1242/jeb.202.20.2859

[B123] Roberts L. H. (1975). The rodent ultrasound production mechanism. Ultrasonics 13, 83–88 10.1016/0041-624x(75)90052-9 1167711

[B124] Sakayori S. Kitama T. Chimoto S. Qin L. Sato Y. (2002). Critical spectral regions forvowel identification. Neurosci. Res. 43, 155–162 10.1016/s0168-0102(02)00026-3 12067751

[B125] Samson S. Zatorre R. J. (1994). Contribution of the right temporal lobe to musical timbre discrimination. Neuropsychologia 32, 231–240 10.1016/0028-3932(94)90008-6 8190246

[B126] Samson S. Zatorre R. J. Ramsay J. O. (2002). Deficits of musical timbre perception after unilateral temporal-lobe lesion revealed with multidimensional scaling. Brain 125, 511–523 10.1093/brain/awf051 11872609

[B127] Sanvito S. Galimberti F. Miller E. H. (2007). Vocal signalling of male southern elephant seals is honest but imprecise. Anim. Behav. 73, 287–299 10.1016/j.anbehav.2006.08.005

[B128] Scharinger M. Idsardi W. J. Poe S. (2011). A comprehensive three-dimensional cortical map of vowel space. J. Cogn. Neurosci. 23, 3972–3982 10.1162/jocn_a_00056 21568638

[B129] Schebesch G. Lingner A. Firzlaff U. Wiegrebe L. Grothe B. (2010). Perception and neural representation of size-variant human vowels in the Mongolian gerbil (Meriones unguiculatus). Hear. Res. 261, 1–8 10.1016/j.heares.2009.12.016 20004713

[B130] Sinnnott J. M. (1989). Detection and discrimination of synthetic English vowels by old world monkeys (Cercopithecus, Macaca) and humans. J. Acoust. Soc. Am. 86, 557–565 10.1121/1.398235 2768672

[B131] Sinnott J. M. Brown C. H. Malik W. T. Kressley R. A. (1997). A multidimensional scaling analysis of vowel discrimination in humans and monkeys. Percept. Psychophys. 59, 1214–1224 10.3758/bf03214209 9401456

[B132] Sinnott J. M. Kreiter N. A. (1991). Differential sensitivity to vowel continua in old world monkeys (Macaca) and humans. J. Acoust. Soc. Am. 89, 2421–2429 10.1121/1.400974 1861002

[B133] Sinnott J. M. Mosqueda S. B. (2003). Effects of aging on speech sound discrimination in the Mongolian Gerbil. Ear Hear. 24, 30–37 10.1097/01.aud.0000051747.58107.89 12598811

[B134] Sinnott J. M. Owren M. J. Petersen M. R. (1987). Auditory frequency discrimination in primates: species differences (Cercopithecus, Macaca, Homo). J. Comp. Physiol. 101, 126–131 10.1037//0735-7036.101.2.126

[B135] Smith D. R. Patterson R. D. Turner R. Kawahara H. Irino T. (2005). The processing and perception of size information in speech sounds. J. Acoust. Soc. Am. 117, 305–318 10.1121/1.1828637 15704423 PMC2346562

[B136] Sommers M. S. Moody D. B. Prosen C. A. Stebbins W. C. (1992). Formant frequency discrimination by Japanese macaques (Macaca fuscata). J. Acoust. Soc. Am. 91, 3499–3510 10.1121/1.402839 1619126

[B137] Staeren N. Renvall H. De Martino F. Goebel R. Formisano E. (2009). Sound categories are represented as distributed patterns in the human auditory cortex. Curr. Biol. 19, 498–502 10.1016/j.cub.2009.01.066 19268594

[B138] Sutter M. L. Schreiner C. E. (1991). Physiology and topography of neurons with multipeaked tuning curves in cat primary auditory cortex. J. Neurophysiol. 65, 1207–1226 1869913 10.1152/jn.1991.65.5.1207

[B139] Swanepoel R. Oosthuizen D. J. J. Hanekom J. J. (2012). The relative importance of spectral cues for vowel recognition in severe noise. J. Acoust. Soc. Am. 132, 2652–2662 10.1121/1.4751543 23039458

[B140] Ter Keurs M. Festen J. M. Plomp R. (1992). Effect of spectral envelope smearing on speech reception. I. J. Acoust. Soc. Am. 91, 2872–2880 10.1121/1.402950 1629480

[B141] Tucker S. Brown G. J. (2003). “Modelling the auditory perception of size, shape and material: applications to the classification of transient sonar sounds,” in 114th Audio Engineering Society Convention (Amsterdam, Netherlands).

[B142] van Dinther R. Patterson R. D. (2006). Perception of acoustic scale and size in musical instrument sounds. J. Acoust. Soc. Am. 120, 2158–2176 10.1121/1.2338295 17069313 PMC2821800

[B143] Vannoni E. Mcelligott A. G. (2008). Low frequency groans indicate larger and more dominant fallow deer (Dama dama) males. PLoS One 3:e3113 10.1371/journal.pone.0003113 18769619 PMC2518835

[B144] Versnel H. Shamma S. A. (1998). Spectral-ripple representation of steady-state vowels in primary auditory cortex. J. Acoust. Soc. Am. 103, 2502–2514 10.1121/1.422771 9604344

[B145] Voigt H. F. Sachs M. B. Young E. D. (1982). Representation of whispered vowels in discharge patterns of auditory-nerve fibers. Hear. Res. 8, 49–58 10.1016/0378-5955(82)90033-8 7142032

[B146] Von Kriegstein K. Smith D. R. Patterson R. D. Ives D. T. Griffiths T. D. (2007). Neural representation of auditory size in the human voice and in sounds from other resonant sources. Curr. Biol. 17, 1123–1128 10.1016/j.cub.2007.05.061 17600716 PMC2335591

[B147] Von Kriegstein K. Warren J. D. Ives D. T. Patterson R. D. Griffiths T. D. (2006). Processing the acoustic effect of size in speech sounds. Neuroimage 32, 368–375 10.1016/j.neuroimage.2006.02.045 16644240

[B148] Walker K. M. M. Bizley J. K. King A. J. Schnupp J. W. H. (2011). Multiplexed and robust representations of sound features in auditory cortex. J. Neurosci. 31, 14565–14576 10.1523/jneurosci.2074-11.2011 21994373 PMC3272412

[B149] Warren J. D. Jennings A. R. Griffiths T. D. (2005). Analysis of the spectral envelope of sounds by the human brain. Neuroimage 24, 1052–1057 10.1016/j.neuroimage.2004.10.031 15670682

[B150] Wedin L. Goude G. (1972). Dimension analysis of the perception of instrumental timbre. Scand. J. Psychol. 13, 228–240 10.1111/j.1467-9450.1972.tb00071.x 5074084

[B151] Wessel D. L. (1979). Timbre space as a musical control structure. Computer Music J. 3, 45–52 10.2307/3680283

[B152] Wier C. C. Jesteadt W. Green D. M. (1977). Frequency discrimination as a function of frequency and sensation level. J. Acoust. Soc. Am. 61, 178–184 10.1121/1.381251 833369

[B153] Young E. D. Sachs M. B. (1979). Representation of steady-state vowels in the temporal aspects of the discharge patterns of populations of auditory-nerve fibers. J. Acoust. Soc. Am. 66, 1381–1403 10.1121/1.383532 500976

[B154] Zahorian S. A. Jagharghi A. J. (1993). Spectral-shape features versus formants as acoustic correlates for vowels. J. Acoust. Soc. Am. 94, 1966–1982 10.1121/1.407520 8227741

